# Role of Thiamine Supplementation in the Treatment of Chronic Heart Failure: An Updated Meta‐Analysis of Randomized Controlled Trials

**DOI:** 10.1002/clc.24309

**Published:** 2024-06-28

**Authors:** Shuai He, Shasha Wang, Tingli Xu, Shuwei Wang, Minfang Qi, Qingqing Chen, Lu Lin, Huijuan Wu, Pengcheng Gan

**Affiliations:** ^1^ Department of Hand and Foot Surgery Taizhou Hospital of Zhejiang Province Affiliated to Wenzhou Medical University Taizhou Zhejiang China; ^2^ Department of Intensive Care Rehabilitation Taizhou Hospital of Zhejiang Province Affiliated to Wenzhou Medical University Taizhou Zhejiang China

**Keywords:** cardiac function, chronic heart failure, thiamine, thiamine deficiency

## Abstract

**Background:**

Chronic heart failure (CHF) has always posed a significant threat to human survival and health. The efficacy of thiamine supplementation in CHF patients remains uncertain.

**Hypothesis:**

Receiving supplementary thiamine may not confer benefits to patients with CHF.

**Methods:**

A comprehensive search was conducted across the Cochrane Library, PubMed, EMBASE, ClinicalTrials.gov, and Web of Science databases up until May 2023 to identify articles investigating the effects of thiamine supplementation in CHF patients. Predefined criteria were utilized for selecting data on study characteristics and results.

**Results:**

Seven randomized, double‐blind, controlled trials (five parallel trials and two crossover trials) involving a total of 274 patients were enrolled. The results of the meta‐analysis pooling these studies did not reveal any significant effect of thiamine treatment compared with placebo on left ventricular ejection fraction (WMD = 1.653%, 95% CI:  −1.098 to 4.405, *p* = 0.239, *I* ^2^ = 61.8%), left ventricular end‐diastolic volume (WMD = −6.831 mL, 95% CI:  −26.367 to 12.704, *p* = 0.493, *I* ^2^ = 0.0%), 6‐min walking test (WMD = 16.526 m, 95% CI:  −36.582 to 69.634, *p* = 0.542, *I* ^2^ = 66.3%), N‐terminal pro‐B type natriuretic peptide (WMD = 258.150 pg/mL, 95% CI:  −236.406 to 752.707, *p* = 0.306, *I* ^2^ = 21.6%), or New York Heart Association class (WMD = −0.223, 95% CI:  −0.781 to 0.335, *p* = 0.434, *I* ^2^ = 87.1%). However, it effectively improved the status of thiamine deficiency (TD).

**Conclusions:**

Our meta‐analysis indicates that thiamine supplementation does not have a direct therapeutic effect on CHF, except for correcting TD.

## Graphical abstract

This meta‐analysis aimed to assess the efficacy of thiamine supplementation on parameters including left ventricular ejection fraction, left ventricular end‐diastolic volume, 6‐min walk test performance, N‐terminal pro‐B‐type natriuretic peptide level, and New York Heart Association class in patients diagnosed with chronic heart failure (CHF). Pooled data analysis did not reveal any significant clinical benefits associated with thiamine intervention. However, it should be noted that thiamine supplementation can effectively address thiamine deficiency in patients with CHF.

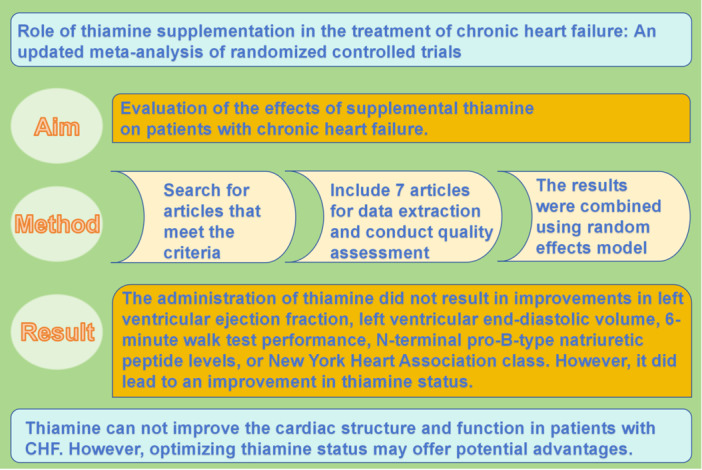

## 1 Introduction

Chronic heart failure (CHF) represents the final stage of various cardiovascular diseases and constitutes a leading cause of mortality, primarily characterized by reduced cardiac output and circulatory congestion [1]. Despite the availability of numerous treatments, the morbidity and mortality rates of patients with CHF continue to rise annually in many countries [2].

Thiamine, also known as vitamin B1, is a water‐soluble vitamin primarily obtained through dietary intake and excreted by the kidneys [3]. Thiamine exists in various forms within the human body and plays a crucial role in numerous essential functions, particularly in cellular energy metabolism [4]. Maintaining an adequate level of thiamine is crucial for the body. Currently, the common method utilized for assessing thiamine levels in clinical practice include measuring the concentration of thiamine pyrophosphate (TPP) in blood [5, 6]. TPP, also known as thiamine diphosphate, is one of the forms in which thiamine exists in the body [4]. In addition, thiamine status can be assessed by quantifying the impact of TPP on erythrocyte transketolase activity, known as TPP effect [7]. Low levels of TPP and a value exceeding 15% of TPP effect all indicate a deficiency in thiamine [6, 8].

Over the past few decades, numerous clinical trials have endeavored to elucidate the role of thiamine in heart failure (HF), yet its precise impact remains elusive [9]. Several earlier studies have suggested that thiamine may be efficacious in improving cardiac function among HF patients [10]. A meta‐analysis conducted previously showed a significant improvement in left ventricular ejection fraction (LVEF) following thiamine supplementation for individuals with CHF [10]. However, with the increasing number of clinical trials conducted on this aspect, the role of thiamine in CHF has been subject to challenge. Recently, a meta‐analysis revealed that thiamine failed to improve outcomes in patients with HF [11]. However, it is noteworthy that this study encompassed both acute heart failure (AHF) and CHF, which are characterized by distinct pathophysiological mechanisms. Therefore, the effectiveness of thiamine in CHF remains uncertain. Furthermore, previous meta‐analyses have not fully incorporated published randomized controlled trials (RCTs) [12, 13]. For these reasons, we aimed to conduct an updated meta‐analysis to comprehensively evaluate the role of thiamine in patients with CHF.

## 2 Methods

Our meta‐analysis adhered to the Updated Preferred Reporting Items for Systematic Reviews and Meta‐Analyses (PRISMA) guidelines [14]. The present meta‐analysis has been duly registered with the International Prospective Register of Systematic Reviews, under the unique registration number CRD42023408247. All data in our study were obtained from published articles, thus exempting us from the requirement of ethical approval.

### 2.1 Search Strategy and Selection Criteria

We searched the Cochrane Library, EMBASE, PubMed, ClinicalTrials.gov, and Web of Science databases for relevant articles up until May 2023. The search strategy included terms such as (thiamin OR vitamin B1 OR aneurin OR thiamine mononitrate) AND (heart failure OR heart decompensation OR cardiac failure OR myocardial failure). In addition to the electronic search, we performed a manual search of the relevant literature, including reviews and references.

Inclusion criteria: RCTs; trials were conducted in patients with a diagnosis of CHF; the intervention group was administered any form of thiamine in comparison to the placebo group; studies that provided sufficient information for both the treatment and control groups.

Exclusion criteria: non‐English publication; abstracts lacking complete results; no interesting results.

### 2.2 Data Extraction and Quality Assessment

Two independent reviewers (P.G. and S.H.) applied predefined criteria to review eligible studies and extract the required data. We have collected the following data: study characteristics, patient numbers, treatment protocols including thiamine type and dosage, follow‐up periods, and outcome measures. The primary efficacy endpoint is the LVEF, with additional endpoints including the left ventricular end‐diastolic volume (LVEDV), 6‐min walking test (6MWT), N‐terminal pro‐B‐type natriuretic peptide (NT‐proBNP), New York Heart Association (NYHA) class, and indicators to assess thiamine status such as TPP and TPP effect. We utilized the Cochrane risk of bias tool to assess the potential for bias in the RCTs that were included in our analysis [15].

### 2.3 Statistical Analysis

Continuous variables were expressed as mean and standard deviation (SD). The 95% confidence intervals (CIs) and standard errors (SE) were transformed into SD using the methods outlined in the Cochrane Handbook for Systematic Reviews of Interventions [14]. The heterogeneity of the results was assessed through Cochran's *Q* test and quantified by the *I* ^2^ statistic. Heterogeneity was considered high when *I* ^2^  > 50% [16]. In the presence of significant heterogeneity, a random‐effects model was utilized; otherwise, a fixed‐effects model was employed. Funnel plots and statistical tests including Egger's and Begger's were conducted to evaluate publication bias. In case of evidence indicating publication bias, the trim‐and‐fill method was applied to assess missing relevant studies and ensure the stability of pooled results. Using Stata software (version 16.0; Stata Corporation, College Station, TX) to conduct all statistical analyzes.

## 3 Results

### 3.1 Search Results

**Figure 1:**
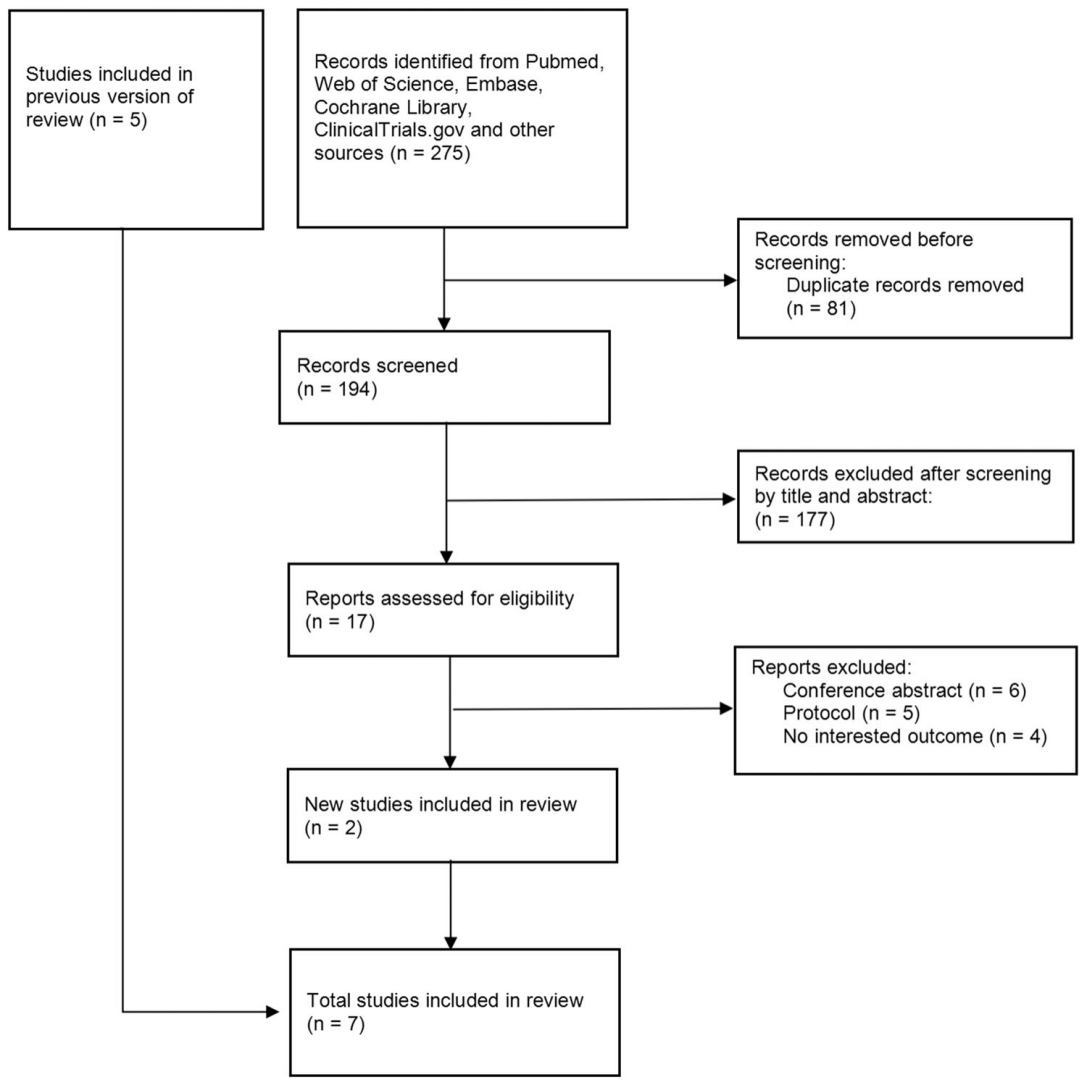
Flow chart of the meta‐analysis.

Five articles were obtained from a previous meta‐analysis [13]. (The authors or institutions involved in this meta‐analysis did not have any overlap with those involved in our study.) A comprehensive search of databases and references yielded 275 eligible articles, which were then screened for duplicates resulting in 194 unique articles. After filtering by title and abstract, 177 irrelevant studies were excluded. The remaining 17 articles underwent full‐text review based on inclusion and exclusion criteria, ultimately leading to the inclusion of two new studies. In the end, a total of seven articles [5, 6, 8, 17, 18, 19, 20] were included in the analysis (Figure 1).

### 3.2 Study Characteristics

Table 1 presents a summary of the clinical characteristics of the studies included in this analysis. All trials, comprising five parallel and two crossover designs, were randomized, double‐blind, placebo‐controlled investigations. In addition to the five studies identified from previous meta‐analysis [13], we conducted a comprehensive search and included two additional studies by Mousavi et al. [17] and Sofi et al. [20], thereby augmenting the participant pool by 92 individuals. Data from 274 individuals were included in the current meta‐analysis, with thiamine doses ranging from 100 to 500 mg/day and treatment durations spanning from 1 week to 6 months.

**Table 1:** Patient characteristics.

Auther (year)	Study design	Country	Male (*n*)	Age, thiamine/placebo (mean ± SD)	Usage and dosage of thiamine	Follow‐up	Outcomes
Iqbal (2019)	Double‐blind	India	27/50	61.4 ± 7.1/62.4 ± 8.0	200 mg/d IV thiamine	6 weeks	LVEF, NYHA, TPPE
Keith (2019)	Double‐blind	Canada	58/69	64 ± 11/63 ± 14	200 mg/d oral thiamine	6 months	LVEF, 6WMT, NT‐proBNP, TPP
Mousavi (2017)^a^	Double‐blind	Iran	35/52	61.92 ± 10.73/60.96 ± 12.94	300 mg/d thiamine	1 month	LVEF
Schoenenberger (2012)	Double‐blind Cross‐over	Switzerland	7/9	56.7 ± 9.2/56.7 ± 9.2	300 mg/d oral thiamine	29 days	LVEF, LVEDV, 6WMT
Shimon (1995)	Double‐blind	Israel	22/30	67 ± 12/72 ± 9	300 mg/d IV thiamine	1 week	LVEF, LVEDV, TPPE
Sofi (2015)^a^	Double‐blind	India	23/40	49.7 ± 7.51/50.45 ± 10.87	100 mg/d oral thiamine	4 weeks	LVEF, LVEDV, 6WMT
Wong (2022)	Double‐blind Cross‐over	Canada	17/24	72.6 ± 7/74.1 ± 8	500 mg/d oral thiamine	3 months	LVEF, NT‐proBNP, NYHA, TPP

Abbreviations: 6MWT, 6‐min walking test; IV, intravenous injection; LVEDV, left ventricular end‐diastolic volume; LVEF, left ventricular ejection fraction; NT‐proBNP, N‐terminal pro‐B‐type natriuretic peptide; NYHA, New York Heart Association; TPP, thiamine pyrophosphate; TPPE, thiamine pyrophosphate effect.

a Additional studies added to the previous meta‐analysis.

### 3.3 Quality Assessment

**Figure 2:**
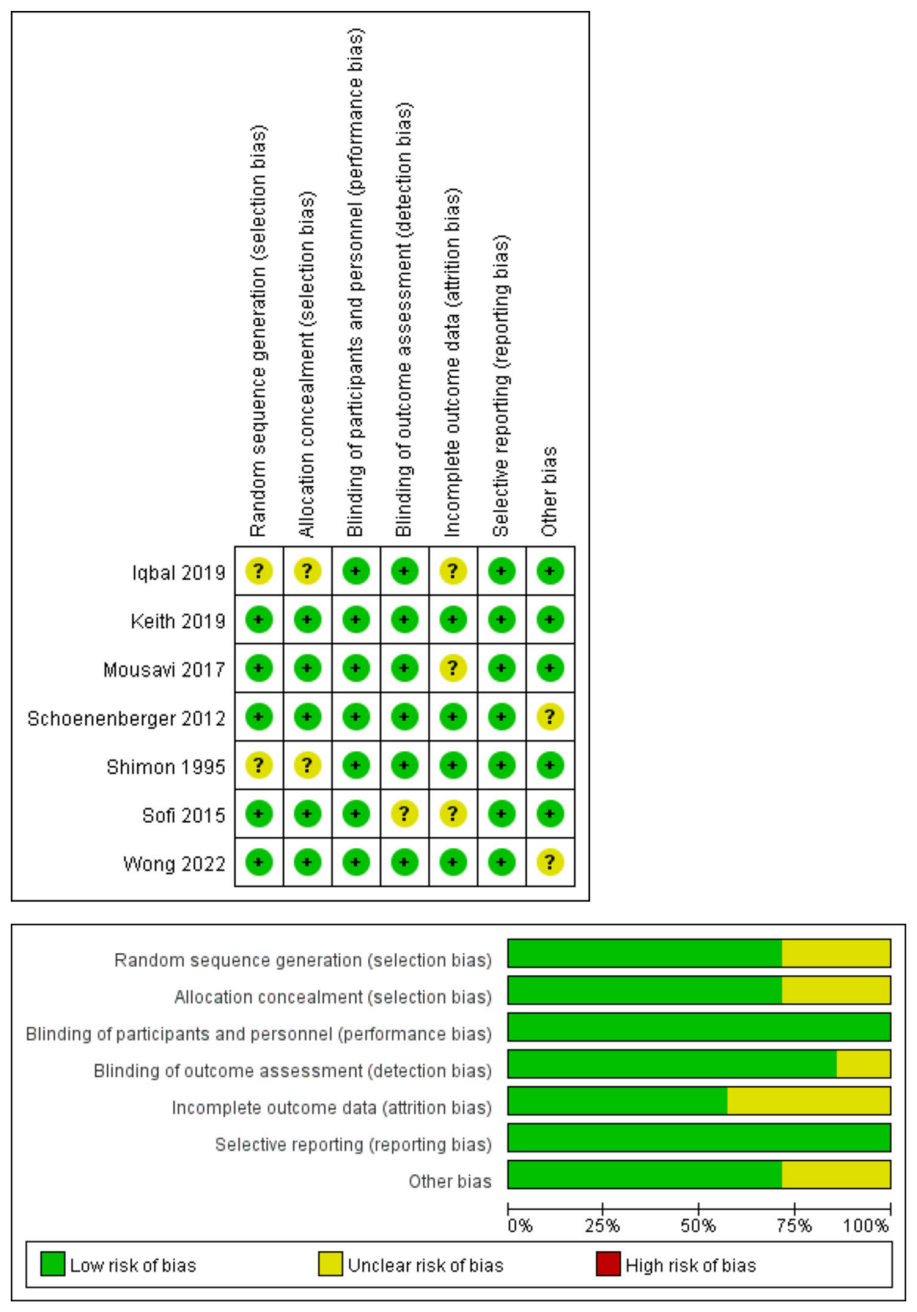
Quality evaluations of randomized controlled trials.

All studies reported blinding of participants and personnel, as well as selective reporting, with a low risk of bias. However, three articles failed to disclose whether the resulting data were incomplete. Additionally, two studies had unclear methods for random sequence generation and allocation concealment. Sofi et al. [20] did not explicitly mention blinding of outcome assessment. Two studies had unclear other biases due to small sample sizes and an unbalanced baseline. The assessment of bias risk is presented in Figure 2.

## 4 Results

### 4.1 Primary End Point

**Figure 3:**
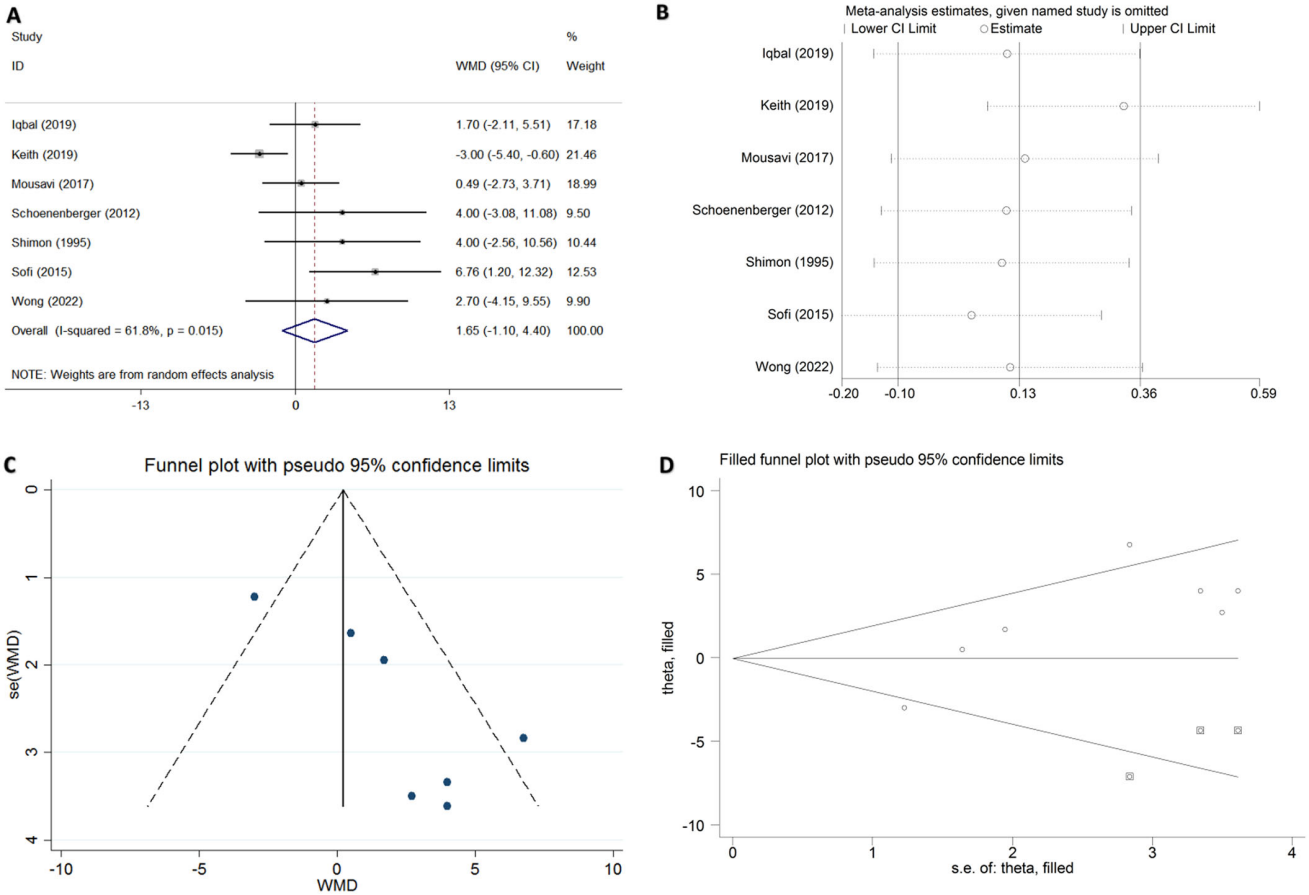
(A) Forest plot summarizing the relationship between thiamine supplementation and left ventricular ejection fraction (LVEF). (B) Sensitivity analysis for the LVEF. (C) Funnel plot of the studies included in the meta‐analysis. (D) Funnel plot using the trim‐and‐fill method. CI, confidence interval; WMD, weighted mean difference.

All studies evaluated LVEF changes during thiamine treatment. Four of the seven studies reported a significant improvement in LVEF after thiamine supplementation compared to placebo [8, 18, 19, 20]. However, the random effects model did not reveal any significant differences between the thiamine and placebo groups in terms of pooled effect sizes (WMD = 1.653%, 95% CI:  −1.098 to 4.405, *p* = 0.239; Figure 3A). A significant level of heterogeneity was observed (*I* ^2^ = 61.8%, *p* = 0.015). Therefore, a sensitivity analysis was conducted (Figure 3B), which revealed that Keith et al. [5] contributed the most to the heterogeneity in this meta‐analysis. The exclusion of this study resulted in a substantial reduction in heterogeneity (*I* ^2^ = 0, *p* = 0.51). Although the heterogeneity was mitigated after excluding the study conducted by Keith et al. [5], we consider this study, with its extensive follow‐up and substantial participant pool, to be of significant impact and should not be excluded.

Using a funnel plot to assess publication bias in the seven included studies, we observed asymmetry in the funnel plot (Figure 3C), indicating the presence of publication bias. Employing Stata version 16.0, Egger's test yielded *p*  = 0.013 and Begg's test yielded *p* = 0.23. There is evidence suggesting that Egger's test may be more sensitive in detecting publication bias compared to Begg's test [21]. A *p* < 0.05 from Egger's test was found to be indicative of publication bias. To further assess this, we employed the trim‐and‐fill method and estimated the presence of three unpublished studies (Figure 3D). After incorporating the three unpublished studies, the funnel plots exhibited symmetry and the newly calculated pooled differences between treatment and placebo groups remained statistically insignificant (WMD = −0.055%, 95% CI: −2.612 to 2.501, *p* = 0.966). As there was no significant alteration in results upon inclusion of these studies, we concluded that publication bias was deemed acceptable.

### 4.2 Secondary End Points

**Figure 4:**
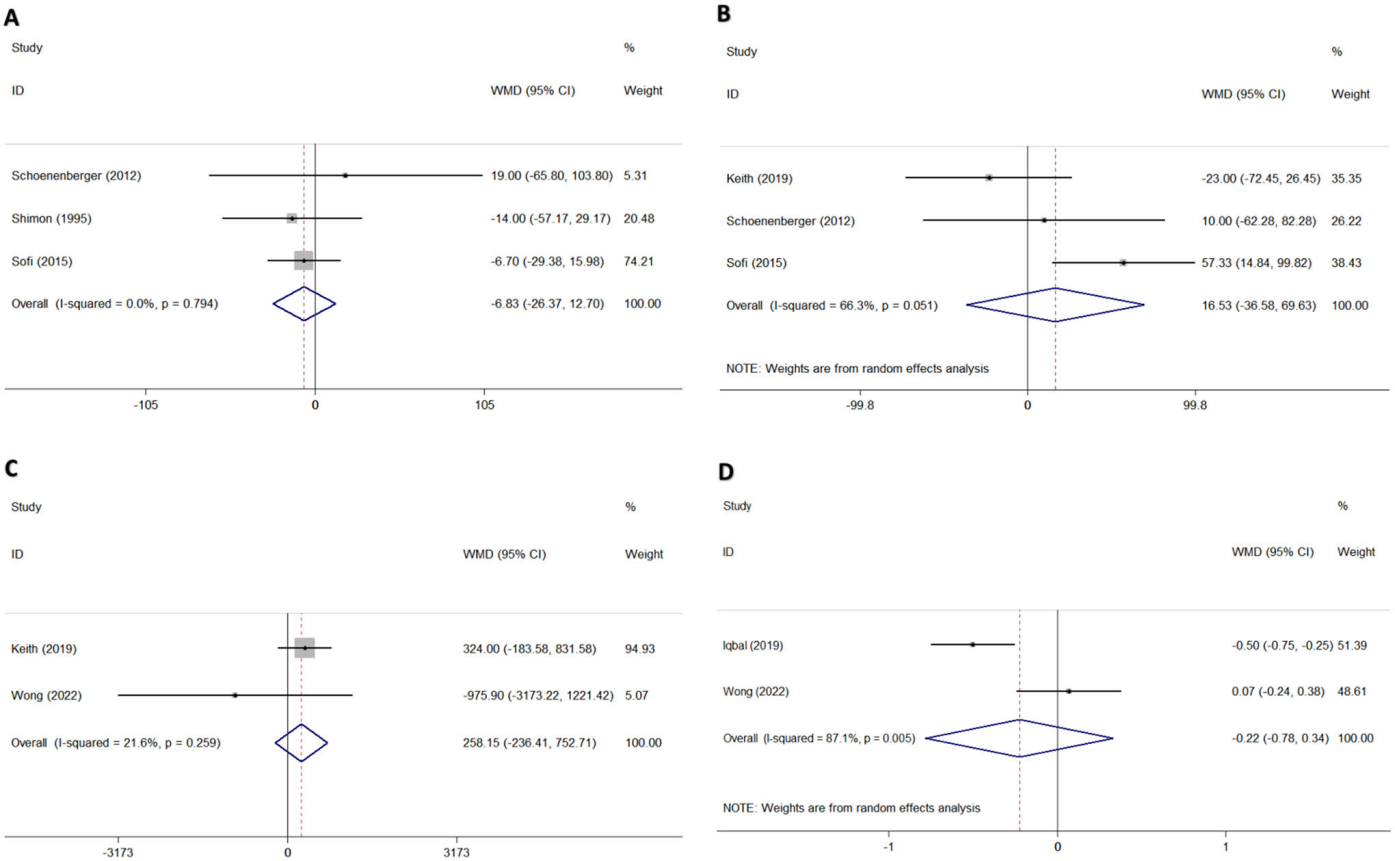
Forest plot summarizing the relationship between thiamine supplementation and (A) LVEDV, (B) 6MWT, (C) NT‐proBNP, and (D) NYHA class. 6MWT, 6‐min walking test; CI, confidence interval; LVEDV, left ventricular end‐diastolic volume; NT‐proBNP, N‐terminal pro‐B‐type natriuretic peptide, NYHA, New York Heart Association; WMD, weighted mean difference.

Three trials [18, 19, 20] provided LVEDV data, none of them demonstrated a significant difference between the thiamine and control groups. The pooled results also indicated that thiamine treatment did not have an impact on LVEDV (WMD = −6.831 mL, 95% CI: −26.367 to 12.704, *p* = 0.493, *I* ^2^ = 0.0%; Figure 4A).

Three articles [5, 18, 20] reported data on the 6MWT. While only Sofi et al. [20] demonstrated a significant improvement in the thiamine group, the pooled results suggested that thiamine supplementation did not enhance performance on the 6MWT (WMD = 16.526 m, 95% CI:  −36.582 to 69.634, *p* = 0.542, *I* ^2^ = 66.3%; Figure 4B).

Two studies [5, 6] evaluated the impact of thiamine treatment on NT‐proBNP levels and found no significant differences compared to placebo. Our meta‐analysis yielded similar results, supporting this conclusion (WMD = 258.150 pg/mL, 95% CI: −236.406 to 752.707, *p* = 0.306, *I* ^2^ = 21.6%; Figure 4C).

Iqbal et al. [8] observed a decrease in NYHA class among the thiamine group compared to the control group, whereas this was not evident in the study by Wong et al. [6]. The combined findings suggested that there was no discernible distinction between the two groups (WMD = −0.223, 95% CI:  −0.781 to 0.335, *p* = 0.434, *I* ^2^ = 87.1%; Figure 4D).

Changes in thiamine status were evaluated in four trials. Shimon et al. [19] observed a significant reduction in TPP effect following thiamine supplementation, with values decreasing from 11.7% ± 6.5% to 5.4% ± 3.2% (*p* < 0.01). Iqbal et al. [8] also reported significantly lower TPP effect levels in the thiamine group compared to the control group (6.8% ± 1.5% vs. 16.0% ± 6.7%, *p* < 0.01). Furthermore, Keith et al. [5] and Wong et al. [6] demonstrate graphically that patients with heart failure who received thiamine had significantly higher erythrocyte TPP concentrations compared to those who received placebo.

## 5 Discussion

We conducted a meta‐analysis that synthesized data from seven RCTs to assess the impact of thiamine supplementation on patients with CHF. In the overall analysis, we concluded that thiamine could not improve the LVEF, LVEDV, NT‐proBNP, 6MWT, or NYHA class in CHF patients. The parameters suggest that thiamine may not possess therapeutic efficacy for CHF; however, an analysis of the results regarding thiamine status reveals that appropriate supplementation could improve thiamine deficiency (TD) or enhance thiamine levels in the body.

In the past, LVEF was the primary indicator of interest in evaluating thiamine treatment for HF. However, a meta‐analysis of five RCTs conducted by Xu et al. [13] revealed that thiamine did not improve LVEF in patients with CHF. In addition to these five studies, we identified two additional double‐blind RCTs through a comprehensive search. Sofi et al. [20] reported no significant improvement in LVEF after 1 week of oral thiamine supplementation among patients with CHF of NYHA class II or III (LVEF < 50%). However, continued thiamine supplementation for 4 weeks resulted in significantly higher LVEF values in the thiamine group compared to the placebo group. The possible explanation for this outcome is that edema in the intestinal wall of HF patients may impede the absorption of oral thiamine, resulting in a delayed alteration in LVEF. However, Mousvi et al. [17] failed to observe any improvement in LVEF after administering thiamine to patients with CHF (LVEF ≤ 40%, NYHA class II) for a duration of 1 month. Our meta‐analysis of seven RCTs also demonstrated that thiamine did not confer any benefit on improving LVEF in patients with CHF. However, our results exhibited some degree of heterogeneity. Upon investigation into the source of this heterogeneity, it was observed that removal of the study conducted by Keith et al. [5] resulted in a significant reduction in heterogeneity (*I* ^2^ decreased from 51% to 0%). Keith et al. [5] employed cardiovascular magnetic resonance (CMR) to assess LVEF in patients diagnosed with CHF, whereas the remaining six studies utilized echocardiography. Research has indicated that CMR images obtained from multiple cardiac cycles may offer greater accuracy in measuring LVEF compared to echocardiography [22]. One possible explanation for the observed heterogeneity is the employment of different methods for assessing LVEF.

A recent meta‐analysis of six RCTs found that thiamine supplementation did not significantly impact the outcomes of patients with HF [11]. However, we have noted that one of the studies included in this analysis employed a combination of high‐dose micronutrients (vitamin A, thiamine, vitamin C, vitamin D, etc.) for treating CHF, with thiamine accounting for only a small fraction [23]. This does not necessarily preclude the possibility that other vitamins may have exerted an impact on treatment outcomes. For instance, certain studies have suggested that vitamin D could confer some ancillary advantages in the management of HF [24]. This concept, however, continues to be a subject of controversy. As such, this particular study was excluded from our analysis.

A noteworthy phenomenon is the high prevalence of TD among patients with HF, with studies indicating that TD affects 21%–98% of such patients [12]. The mechanism behind this remains unclear. Previous research has suggested that loop diuretics—commonly prescribed for HF—may contribute by increasing urine flow [25, 26]. In addition, cell experiments have demonstrated that furosemide, a type of loop diuretic, can impede thiamine uptake by cardiac cells via its impact on the sodium gradient [27]. Moreover, multiple lines of evidence have demonstrated that malnutrition resulting from various etiologies and heightened metabolism are also significant contributors to TD in patients with HF [28].

Additionally, TD exerts significant effects on the cardiovascular system. For example, TD can result in wet beriberi, which primarily affects myocardial energy metabolism and reduces peripheral vascular resistance. This leads to high‐output HF, characterized by cardiac enlargement, edema, and increased venous pressure [29]. Thiamine supplementation is an effective treatment for wet beriberi and can lead to significant improvement in clinical symptoms. However, our study did not reveal any significant improvement in cardiac function (LVEF) or heart size (LVEDV) even after adequate thiamine supplementation in CHF patients with prevalent TD. Therefore, we speculate that CHF is a multifactorial clinical syndrome and thiamine supplementation alone may not be sufficient for effective treatment. However, it may be beneficial in cases of HF primarily caused by TD. In addition, correcting TD may confer benefits in terms of preventing further cardiac function deterioration and reducing the incidence of other complications in patients with CHF. For example, a recent study demonstrated that thiamine supplementation significantly decreased in‐hospital mortality rates among severe HF patients admitted to the intensive care unit [30].

Currently, the specific reference intake and method of thiamine supplementation for patients with TD remain controversial. Initially, it was believed that oral administration had low bioavailability and was ineffective in improving TD [31]. However, recent evidence suggests that oral thiamine is still effective and has fewer side effects than intravenous administration [29]. Given the guidelines and consensus recommending thiamine supplementation for HF patients [32, 33], we deem timely monitoring of thiamine status in CHF patients to be pivotal, as it can provide enhanced guidance on the utilization of thiamine.

The limitations of our study need to be acknowledged. First, the sample size and participant number were limited, and some studies lacked a sufficiently long follow‐up period. Second, we combined both parallel trials and cross‐over trials in our meta‐analysis; however, there is still ongoing debate regarding the combination of these two types of trial in meta‐analyses. Third, this study specifically investigated the impact of thiamine on patients with CHF and was not further analyzed due to limited research on its correlation with AHF. Moreover, publication bias was detected in the assessment of LVEF. Although the trim‐and‐fill method limited to seven studies suggested that the pooled results were robust, more large‐scale trials are needed due to the limited evidence.

## 6 Conclusion

In this meta‐analysis of seven RCTs involving 274 patients, we did not observe a curative effect of thiamine on patients with congestive HF when comparing changes in LVEF, LVEDV, NT‐proBNP, 6MWT, and NYHA class. However, improving TD status may be potentially beneficial in CHF. Appropriate thiamine supplementation is still necessary for patients with CHF and more robust and long‐term RCTs are needed to further clarify our conclusions.

## Conflicts of Interest

The authors declare no conflicts of interest.

## Data Availability

Data available upon request from the corresponding author.
